# Circulating Biomarkers for Cancer Detection: Could Salivary microRNAs Be an Opportunity for Ovarian Cancer Diagnostics?

**DOI:** 10.3390/biomedicines11030652

**Published:** 2023-02-21

**Authors:** Marzia Robotti, Francesca Scebba, Debora Angeloni

**Affiliations:** 1 Graduate School in Translational Medicine, Scuola Superiore Sant’Anna, Via G. Moruzzi, 56124 Pisa, Italy; 2 Health Science Interdisciplinary Center, Scuola Superiore Sant’Anna, Via G. Moruzzi, 56124 Pisa, Italy; 3 The Institute of Biorobotics, Scuola Superiore Sant’Anna, Via G. Moruzzi, 56124 Pisa, Italy

**Keywords:** ovarian cancer, biomarker, saliva, urine, ascites, blood, microRNA, biofluids, early diagnosis, screening

## Abstract

MicroRNAs (miRNAs) are small non-coding RNAs with the crucial regulatory functions of gene expression at post-transcriptional level, detectable in cell and tissue extracts, and body fluids. For their stability in body fluids and accessibility to sampling, circulating miRNAs and changes of their concentration may represent suitable disease biomarkers, with diagnostic and prognostic relevance. A solid literature now describes the profiling of circulating miRNA signatures for several tumor types. Among body fluids, saliva accurately reflects systemic pathophysiological conditions, representing a promising diagnostic resource for the future of low-cost screening procedures for systemic diseases, including cancer. Here, we provide a review of literature about miRNAs as potential disease biomarkers with regard to ovarian cancer (OC), with an excursus about liquid biopsies, and saliva in particular. We also report on salivary miRNAs as biomarkers in oncological conditions other than OC, as well as on OC biomarkers other than miRNAs. While the clinical need for an effective tool for OC screening remains unmet, it would be advisable to combine within a single diagnostic platform, the tools for detecting patterns of both protein and miRNA biomarkers to provide the screening robustness that single molecular species separately were not able to provide so far.

## 1. Introduction

We reviewed the current literature to understand what opportunities exist in terms of circulating microRNA (miRNA), specifically salivary miRNAs, as biomarkers (BMs) for diagnostic/prognostic approaches to oncological diseases in general, and to ovarian cancer (OC) in particular. We realized that currently there are not miRNA BMs in clinical use for OC screening.

First, we provide a general introduction on the subject of OC and the state of the art in terms of screening opportunities. Section 2 introduces the subject of miRNAs, showing their recognized potential as disease biomarkers (BM). In Section 3, we analyze relevant literature on the potential of liquid biopsies for the detection of miRNAs in OC, with a specific focus on saliva (Section 4) to discuss the pros and cons of this biofluid, whose molecular profile resembles blood, with possible advantageous simplifications. For the sake of completeness, and to support the hypothesis that the search for salivary miRNAs could be useful in OC, we also reviewed the state of the art of salivary miRNAs in oncological conditions other than OC (Section 5), and, to support the validity of saliva as a useful biofluid for BMs search, we provide an excursus (Section 6) on OC candidate salivary BMs other than miRNAs. Finally, to compare saliva with other possible biofluids as a source of miRNA BMs, we reviewed the literature on this type of candidate BMs of OC in other biofluids (Section 7).

Overall, this review aims to provide an informed understanding of the need for more research on the subject of reliable BMs for OC screening, because presently, the clinical need for an early, reliable, relatively inexpensive diagnostic tool for OC screening in the population remains unmet. Specifically, we propose saliva as a convenient bioanalyte, possibly for the combined search of candidate BMs of different nature, such as miRNA and proteins.

OC, although rare, is the first cause of death for gynecological cancers and the fifth most common cause of cancer-related death for women of industrialized countries [1]. The poor outcome is mostly associated with the lack of specific symptoms and of effective screening strategies. Despite increasingly radical surgical approaches and huge efforts put into new, targeted, therapeutic agents, the prognosis for patients with OC has hardly improved in the past three decades and two thirds of women still die within ten years from diagnosis [2]. Five-year survival is less than 20% in women diagnosed with advanced-stage (stage III or IV) invasive epithelial OC, but exceeds 90% in those detected at stage I [3], suggesting that success in curing OC relies on early diagnosis. Efforts have therefore focused on diagnosing early-stage or low-volume disease through risk prediction, prevention, and screening. Worldwide, there is inconsistency in availability of and access to treatment for OC, and the outcomes are complicated because of the complexities of the disease in terms of epidemiology, genetic features, and histopathology, all contributing to the still poor understanding of OC, especially in comparison with other oncological diseases [4].

In fact, OC is heterogeneous, comprising several disease types and subtypes [5,6]. The extra-ovarian originations of epithelial OCs contribute to the intricacies of the disease. Over the past decade, a dualistic pathway of epithelial ovarian carcinogenesis has emerged: Type I and Type II, that differ in epidemiology, etiology, and treatment. Therapeutic strategies may not be equally effective for all. Therefore, success in treating OC also requires to identify new BMs, not only to detect OC early, at a time when outcomes could be improved, but also to allow a better stratification of patients with full blown disease for more efficacious, personalized therapy [7,8,9].

Years ago, the first biomarker (BM) identified and proposed as a blood test for women with OC was the carcinoembryonic antigen (CEA) [10]. However, the current literature agrees that multi-BM panels might perform better than a single BM for a personalized treatment in the context of precision medicine in general [9,11] and for early detection of OC in particular [12].

At present time, despite decades of international efforts to identify panels of blood-borne BMs suitable for screening, no one superior to serum cancer antigen 125 (CA125) has reached clinical practice, except for human epididymis protein 4 (HE4), which is still second best to CA125 [13]. Their combined analysis within the risk of ovarian malignancy (ROMA) algorithm has improved the diagnostic accuracy for OC. However, ROMA is not applicable in screening procedures for early detection [9,13,14]. In addition to ROMA, the OVA1 test, a multivariate in vitro assay for five proteomic BMs, is used as an aid to the pre-chirurgical definition of a pelvic mass, but, again, it does not possess the characteristics to be applied on large-scale, prevention screening [15].

Currently, the combination of CA125 serum analysis and transvaginal ultrasonography (TVS) is the most utilized tool for the initial evaluation of suspect cases, even though CA125 lacks specificity (as it rises due to several physiological and pathological conditions in addition to cancer [16]), and TVS can identify adnexal masses, but is less reliable in differentiating benign from malignant tumors [17].

Over time, other serum BMs have been evaluated in OC together with CA125 [18], and several strategies have been developed by combining the analysis of multiple circulating proteins [19,20].

The literature shows that proteins expressed in OC cells can be retrieved in serum and urine [11,21]. However, only a few of several reported candidate BMs were validated for clinical practice [22], this discrepancy being ascribed to different reasons [11,23].

Therefore, regardless of the enormous progress in analytical techniques, the insights on the biochemical and molecular mechanisms of OC and the description in the literature of several candidate BMs, up to now, the clinical need for an effective test able to perform early, reproducible, noninvasive, and possibly inexpensive diagnosis of OC remains completely unanswered [24].

Moreover, despite recent encouraging data on sensitivity and cost-effectiveness of multimodal analysis, screening for OC in the general population is not recommended due to the lack of a definitive mortality benefit. This has been reinforced in the latest recommendation from the U.S. Preventative Task Force (USPSTF) [25] and the U.K. National Screening Committee (UK NSC) [4].

Several recent studies aim at exploring other possible sources of BMs in addition to blood as analyte, and to proteins as BMs. For example, there are studies dealing with tumor DNA detection, also for methylation profiling, in liquid cytology samples from vagina and endocervix, including routinely collected cervical screening specimens, vaginal self-swab and tampons, and uterine lavage samples. Moreover, improvements in imaging methods are pursued to include refinements of TVS, Doppler flow, microbubble contrast-enhanced TVS, and photo-acoustic imaging, all of which allow high-resolution detection of angiogenesis with the potential to detect neovascularization in early cancers [26,27,28]. In addition, multi-omics technologies (genomics, transcriptomics, proteomics, and metabolomics) hold the possibility of discovering new informative BMs [29,30] in signature panels. Currently, important questions in this field are concerned with the type of sample source selected, the analytical technique that leads to the most valuable results, whether it is advisable to look for one specific BM type (e.g., proteins or nucleic acids) or whether to combine the results of different analytical procedures (e.g., proteins and nucleic acids in parallel).

In summary, while on one hand the state of the art in OC screening recommends against screening for fear of adverse complication in false-positive women undergoing surgery, on the other hand, personalized medicine requires an individualized approach and reliable tools for screening procedures to detect the disease at an early, curable stage, to improve patient stratification and therapeutic regimes, and to spare unnecessary, even possibly hazardous treatments and save lives and costs, as invoked by authoritative editorials [31,32].

## 2. MicroRNAs as Potential Disease Biomarkers

**Figure 1:**
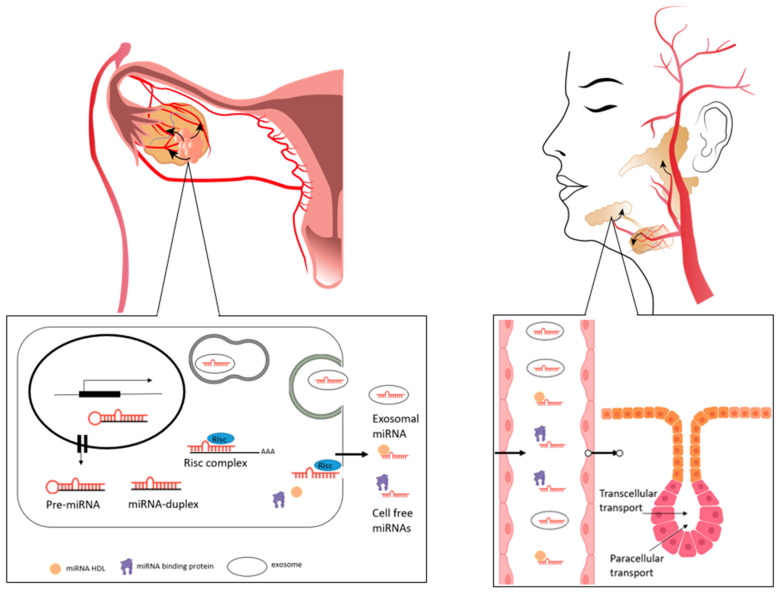
Anatomical and molecular visualization of miRNAs trafficking between OC tissue to bloodstream and saliva. Direction of molecular trafficking between ovarian tissue and bloodstream (**upper left**), and between blood and salivary glands (**upper right**) is indicated by arrows. There is an intense exchange of molecules from the bloodstream to the ovary and vice versa: the ovary is highly vascularized so that it is supplied with hormones and nutrients needed for its high metabolism. At the same time, small molecules, such as microRNAs, are released from the tissue, also under tumor conditions. The composition of blood therefore influences salivary composition. The salivary fluid is produced by the local vascular bed in the acinar region and through the duct system released into the oral cavity. At cell level (**lower left**), miRNAs generated in the cell are released within exosomes or associated with miRNA binding proteins (such AGO2) or high-density lipoprotein (HDL). They are stable and protected by RNase degradation. Intra- and extracellular mechanisms allow these molecules to be transported from blood to saliva (**lower right**), so they can be found in the oral cavity reflecting a distant physiological or pathological condition.

Among molecules with potential BM characteristics, miRNAs have recently emerged as powerful keys to a better understanding of molecular mechanisms controlling cancer cells and their niche. MiRNAs are small molecules (18–25 nucleotides) of single-strand, non-coding RNA (ncRNA) that are found in the transcriptome of all living beings and regulate gene expression at the transcriptional and post-transcriptional level [33] (Figure 1, lower part).

The first evidence that miRNAs may have diagnostic and therapeutic potential was presented soon after the identification of the first miRNAs in humans [34]. The study showed correlation between the loss of miR-15 and miR-16 and the occurrence of B-cell leukemia [35]. Three years later, the homozygous deletion of the gene coding for Dicer, an enzyme essential for miRNA biogenesis, showed that miRNA loss of function disrupted prenatal development of the murine embryo [36], providing the first genetic evidence of miRNAs’ vital role in development. The first indication that miRNAs may become easily accessible BMs for cancer diagnosis and prognosis came three years later when miRNAs were isolated from patient serum [37,38] and their profiling revealed specific patterns across different groups of diseases [39]. Following studies confirmed specific miRNA signatures in many types of human diseases, including different cancers [40,41,42]. MiRNA signature in blood is comparable to that of the tumor of origin [43] and specific miRNAs were associated with prostate cancer (PrC) [38], lung cancer (LC), colorectal (CRC) [44,45,46], breast cancer (BC), gastric cancer (GC) and OC [47,48,49]. Indeed, miRNA profiling revealed different patterns in different histological tumor subtypes of OC, a highly heterogeneous disease, as already mentioned [23,49,50,51,52,53,54].

Several clinical trials were initiated, based on the suggested applicability of miRNAs for cancer diagnostics and prognostics [55]. In fact, miRNAs meet most of the criteria for being ideal BMs, i.e., accessibility, specificity, and sensitivity.

Despite the lack of standardized protocols and use in current clinical practice, miRNAs hold the potential to become a routine approach in the development of personalized medicine in the future.

## 3. Liquid Biopsies for Detection of miRNAs in Ovarian Cancer

Tissue biopsy is the gold standard to evaluate molecular features of tumors. However, in the case of OC, tissue biopsies should be avoided because puncture can cause cancer cells dissemination into the peritoneal cavity, promoting peritoneal metastasis. On the contrary, liquid biopsy represents a safe choice, also enabling serial sampling over time [29]. Body fluids contain several types of molecules: circulating tumor cells, circulating nucleic acids, and extracellular vesicles. As miRNAs pass from tissues to blood and are stable in body fluids [56], circulating miRNAs are suitable BMs for OC detection and staging [57,58,59,60], (Figure 1, lower part).

Circulating miRNAs can be contained within extracellular vesicles (EVs), which are released by normal and tumor cells and, depending on their size, divided into three main groups: exosomes, microvesicles, and apoptotic bodies [61]. Cancer-derived exosomal miRNAs have captured the interest of many researchers for their role in promoting cancer through angiogenesis and metastasis. Cancer cells may produce more exosomes than normal cells; for this reason, exosomes could be informative regarding patients’ health [62].

However, miRNAs can also be found in the EV-free fraction, where they are complexed to RNA-binding proteins, lipids, or lipoproteins, in extracellular fluids [63].

Both types of miRNAs are resistant to RNase degradation, temperature, and pH changes [38].

## 4. Saliva: An Informative Analyte for miRNA Detection, with Practical Advantages

Among body fluid analytes, saliva offers some yet unexplored advantages. Saliva collection can be carried out without medical intervention and does not present risks associated with even minimally invasive procedures; the sample, properly stabilized, can be stored and shipped from even remote collection sites to high-tech testing sites with minimal costs. Saliva released by the major salivary glands consists of 99% water containing inorganic and organic species including secretion and putrefaction products, lipids, over 2400 proteins, metabolites, components of the microbiome and abundant, stable extracellular coding- and non-coding RNA species, among which are a wide variety of miRNAs [64]. Saliva represents blood very faithfully, even with possible advantages. For example, storage is simpler; saliva does not coagulate, it is stable for 24 h at room temperature and for a week at 4 °C. In addition, it may be collected at no risk several times a day to monitor therapy with repeated sampling [65]. All the above makes saliva a relatively simple, accurate, easy, safe, and economical material to be tested for clinically significant molecules.

Some of the molecules characterized in saliva are candidate BMs for cancer diagnosis, prognosis, drug monitoring, and pharmacogenetic studies, although just a few of such candidates were validated in multicenter studies, with large sample size and standardized protocols [66].

The salivary transcriptome has been entirely characterized in 2012 [67]. It includes long and small RNA species. Salivary miRNAs are more stable and discriminatory than mRNAs. They are abundant, and fit the profile of other body fluids [68,69], which makes them good BM candidates for systemic diseases. In fact, MiRNAs associated with CRC, esophageal, and pancreatic cancer (PC) were isolated from saliva [70,71], showing that profiling miRNA expression could allow for the detection of a distant neoplasia while discriminating between different cancer types [23,72]. For all these reasons, several current research efforts are focused on the detection of salivary miRNAs, although we need to better understand their biogenesis and how to recognize their tissue of origin [73].

### Kits for Salivary Diagnostics

Indeed, several diagnostic kits that use saliva as the biofluid test have already been developed and are commercially available. The kits encompass different uses; SARS-CoV-2 has received emergency use authorization from the U.S. Food and Drug Administration (FDA) in the last two years, and kits for the search in oral fluids of other viruses such as HIV, HPV, HSV, or other infectious agents (e.g., Candida albicans) are also available (The ADA Science & Research Institute, LLC (ADASRI) for Oral health). Lin-Zhi International, Inc. (LZI) (https://www.lin-zhi.com/oral-fluid-eia, accessed on 20 February 2023) is a manufacturer of in vitro diagnostic reagents for oral fluid screening (in addition to urine) for the detection of drugs of abuse. Salivary miRNA diagnostic tests for ASD (autism spectrum disease) have also been validated (NIH ClinicalTrials.gov, https://www.clinicaltrials.gov/ct2/show/NCT05418023, accessed on 20 February 2023). Salimetrics, LLC (https://salimetrics.com/assay-kits/, accessed on 20 February 2023) produces salivary ELISA kits (hormones, COVID, cytochines) and Salignostics (https://www.salignostics.com/saliva-diagnostics/, accessed on 20 February 2023) produces approved salivary tests for COVID and pregnancy and is developing tests for cardiac risk, malaria, and Helicobacter pylori.

## 5. Salivary miRNAs in Oncological Conditions

To show the overall interest and potential of salivary miRNA in disease detection well beyond pathologies of the oral cavity [74], in this section, we provide the state of the art regarding microRNA dysregulation detected in saliva in association with systemic oncological conditions. With this objective, we reviewed articles published in the last ten years (2012 to January 2023), including case control or cohort studies for diagnostic biomarkers, review article, and meta-analysis. We did not set, as a criterion, a minimum number of patients recruited in the study. We considered only miRNAs associated to cancer types with the primary site distant from the oral cavity; therefore, studies evaluating deregulated miRNAs in patients with pharyngeal, esophageal, salivary glands, and oral cavity diseases were excluded. Similarly, studies concerning neurodegenerative diseases, microbial infections, and drug/hormone response therapy were excluded. Studies on salivary miRNAs for forensic purposes were excluded as well.

Multiple papers highlight qualitative or quantitative differences in miRNA composition between different body fluids [75,76]. At the same time, specific miRNAs are found in all body fluids analyzed. There are some inconsistencies among papers dealing with salivary miRNAs in the same oncological condition, and this can be ascribed to different reasons: the studies were performed on relatively small sample sizes, methods were often different, and subject groups were not always overlapping. Nevertheless, some miRNAs were pointed out by different authors as putative BMs for the same or for different oncological conditions [75,77,78,79].Overall, literature agrees on the promising potential of salivary miRNAs as BMs for cancer detection [80].

PC, the seventh leading cause of cancer-related deaths worldwide, is the most studied in terms of salivary miRNAs. Similar to OC, early-stage PC rarely causes any symptoms, and for this reason it remains one of the most undiagnosed and lethal malignant neoplasms [23]. Carbohydrate antigen 19-9 (CA19-9) is routinely used for prognosis and to monitor the disease [81]; however, it often fails to detect precancerous or early-stage lesions because of inadequate sensitivity and specificity. Therefore, for better prevention or treatment of PC, just as for OC, we have the urgent need to find new technologies for earlier detection. The search for miRNA biomarkers is advancing, although, to date, none has yet reached clinical applications [81]. Papers dealing with salivary miRNAs BMs for PC [75,76,77,82,83], CRC [78,84], LiC [79,85], LC [86], BC [87], GC [88,89], and PrC [90,91] are summarized in Table 1. MiRNAs were detected both as cell free or as exosomal miRNAs, both in saliva alone as well as in other parallel biofluids. Different techniques have been utilized for the discovery phase (microarrays or sequencing) and real-time quantitative PCR (QRT-PCR) was used for validation. Panels of up- or down-regulated miRNAs have been evaluated for their diagnostic ability; in some cases, sensitivity and sensibility have reached high scores (Table 1). Xie et al., 2014, profiled with a miRNome microarray 2006 mature miRNA sequences from saliva samples of 8 patients with resectable PC, and of 8 healthy subjects; they validated significant candidates with QRT-PCR on the same discovery sample set, and finally analyzed the expression level of 10 selected miRNAs on an independent cohort of 40 patients with PC, 20 with benign pancreatic tumors (BPT), and 40 healthy controls. They showed that miR-3679-5p is significantly downregulated and miR-940 significantly upregulated, in PC versus BPT, and in PC versus BPT and controls. No significant differences were observed between BPT and healthy controls [82]. Humeau et al., 2015, reported a salivary miRNA signature for PC, including miR-21, miR-23a, miR-23b, and miR-29c, that was significantly overexpressed in PC patients compared to healthy subjects. MiR-210 and let-7c were up-regulated in pancreatitis patients compared to controls; miR-216 was upregulated in PC compared to pancreatitis. The authors xenografted human-derived PC cells in athymic mice and found that miR-21 was higher in saliva from tumor-bearing mice, while the other three remained low [77]. Machida et al., 2016, analyzed the expression of miR-1246, miR-3976, miR-4306, and miR-4644 (previously reported as PC BMs in serum exosomes [83]) in salivary exosomes of pancreatobiliary tract cancer patients. MiR-1246 and miR-4644 were significantly over-expressed in salivary exosomes of pancreatobiliary tract cancer patients compared to controls [92].

The last two publications regarding salivary miRNAs in PC highlight an important discrepancy to take note of. The level of miR-1246, analyzed in serum, urine, and saliva, was significantly higher in the serum and urine of cancer patients as opposed to healthy subjects, but salivary levels did not differ significantly between the two groups [75]. Similarly, the analysis of six miRNAs (miR-21, miR-155, miR-196a, miR-200b, miR-376a, and miR-34a) in serum and saliva samples showed that, although miR-21 and miR-34a were differentially expressed in serum samples of pancreatic ductal adenocarcinoma patients, and the same miRNAs were detectable in saliva, their levels did not change significantly among cancer patients and controls [76]. These studies were performed on relatively small sample sizes and only one ethnic group was included. As already mentioned for OC studies, methods are still not adequately standardized for miRNA research in PC.

Excluding the pathologies of the oral cavity and PC, other systemic conditions were investigated for miRNA representation in saliva [74]. Sazanov et al., 2017, focused on the expression miR-21 in blood and saliva samples of patients with distal colorectal cancer (CRC) at different stages. It was found that miR-21 was higher in CRC patients than controls, with diagnostic sensitivity and specificity in saliva higher than in blood [84]. Rapado-González et al., 2019, analyzed only saliva samples of CRC and adenoma patients and of healthy subjects. Through validation phases, the authors selected five miRNAs upregulated in CRC compared to healthy controls (Table 1). Building a COX regression analysis with the clinico-pathological parameters, they found that the high level of the five miRNAs, and the concentration of CEA protein, correlated with a higher risk of progression [78]. In liver cancer (LiC), Petkevich et al., 2021 compared the exosomal with the exosomal-free fraction of blood or saliva. They included HCV-related liver cirrhosis, primary liver cancer patients, and healthy volunteers and confirmed in saliva, as already found in plasma, the differential expression of three miRNAs in liver cancer (LiC) patients compared to controls. (Table 1). The saliva exosomal fraction was found to be the best performing [79]. Mariam et al., 2022, with an RNA-seq study in saliva based on 20 hepatocellular cancer (HCC) and 19 cirrhosis patients, found that 283 salivary miRNAs were significantly downregulated in HCC. Machine learning identified a combination of 10 miRNAs as good indicators of LiC. Of interest here, mir-92b, mir-548i-2, and mir-548l were found differentially expressed both in saliva and in HCC tissue samples compared to cirrhotic liver tissue samples [85].

LC, BC, PrC, and GC are the four other oncological conditions for which salivary microRNAs have been investigated. Yang et al., 2020, profiled salivary miRNAs in four groups of lung cancer patients: 57 patients with malignant pleural effusion (MPE); 33 patients with benign pleural effusion (BPE); 50 patients with a diagnosis of malignancy without pleural effusion (MT), and 49 healthy controls (HCs). The discovery phase with microarray was performed on six salivary samples from three MPE patients and three HCs and showed 29 upregulated and 48 downregulated miRNAs in MPE compared to controls. The validation phase performed with QRT-PCR highlighted two miRNAs to be up- and down-regulated in MPE patients respectively, (Table 1) [86]. Koopaie et al., 2021, analyzed with QRT-PCR, 41 BC patients and 39 healthy subjects. MiR-21 was significantly upregulated in BC samples relative to controls, but not among disease stages [87]. Li F. et al., 2018, analyzed salivary samples of 10 early-stage GC patients and 10 non-GC controls, with TaqMan Human miRNA array. Four miRNAs (Table 1) made the best BM panel [88].

Nanographene Oxide (nGO), a novel method to detect circulating oncomiRs, was used by Hizir et al. in PrC. The authors analyzed exogenous miR-21 and miR-141 in saliva and other body fluids using the two-dimensional surface of nGO. The two miRNAs reflected the disease stage: miR-141 was higher in advanced PrC, while miR-21 was higher in early-stage cancer. The copies of circulating miRNAs in patient specimens are lower than the concentration used in this study, thus, the sensitivity of the assay needs to be improved. However, the method is fast and easy, can be performed with a spectrofluorometer, and potentially allows the reveal of PrC patients at different stages with a non-invasive approach [90].

**Table 1:** Salivary miRNAs in oncological conditions. PC: pancreatic cancer, BPT: benign pancreatic tumor, PBTC: pancreatobiliary tract cancer, HS: Healthy subjects, CRC: colorectal cancer, LiC: liver cancer, HCC: epatocellular carcinoma, MPE: malignant pleural effusion, BC: breast cancer, GC: gastric cancer. Each row provides information regarding the citation in parenthesis at the far left end of the row.

Reference	Source (Analytes)	miRNAs	Cancer Type	N° of Subjects (Cancer vs Control)	Status	Sensitivity	Specificity	AUC
[82]	cell free	miR-3679-5p	PC	40 vs 40	↓	72.5%	70.0%	0.750
		miR-940			↑			
[77]	cell free	miR-21	PC	7 vs 4	↑	71.4%	100%	-
		miR-23a			↑	85.7%	100%	-
		miR-23b			↑	85.7%	100%	-
		miR-29c			↑	57.0%	100%	-
[92]	exosomes	miR-1246	PBTC	14 vs 13	↑	83.3%	92.3%	0.833
		miR-4644			↑			
[75]	cell free	miR-1246	PC	41 vs 30	-	91.0%	26.7%	0.480
[84]	cell free	miR-21	CRC	34 vs 34	↑	97.0%	91.0%	-
[78]	cell free	miR-186-5p	CRC	51 vs 37	↑	72.0%	66.7%	0.754
		miR-29a-3p			↑			
		miR-29c-3p			↑			
		miR-766-3p			↑			
		miR-491-5p			↑			
[79]	exosomes cell free	miR-21-5p	LiC	24 vs 21	↑	66.0%	78.0%	0.770
		miR-122-5p			↓			
		miR-221-3p			↑			
[85]	cell free	mir-1262 miR-1262 mir-216a mir-484 mir-30d miR-216a-5p miR-30d-5p miR-484 mir-10401 miR-454-3p	HCC	20 vs 19 Cirrhosis	-	83.0%	68.0%	0.780
[86]	cell free	miR-4484	MPE	57 vs 49	↑	82.2%	74.1%	0.802
		miR-3663-3p			↓			
[87]	cell free	miR-21	BC	41 vs 39	↑	100%	100%	1.0
[88]	cell free	miR-140-5p	GC	100 vs 100	↑	-	-	0.700
		miR-374a			↑	-	-	0.650
		miR-454			↑	-	-	0.630
		miR-15b			↑	-	-	0.650

## 6. Candidate Salivary Biomarkers of Ovarian Cancer Other Than miRNAs

The urgency of finding reliable BMs for early OC detection has prompted many researchers to focus on saliva as an advantageous biofluid in terms of collection, procedure, and cost-effectiveness [93]. Several studies compared saliva with other solid or liquid biopsies for the sake of methodological cross validation, to evaluate the use of saliva as the analyte of choice.

Lee et al., 2012, explored mRNAs expression in saliva of OC patients. The study design was based on: (i) an initial profiling with microarray assay; (ii) validation with QRT-PCR of the preliminary findings obtained on a discovery cohort and on an additional, independent cohort; and (iii) logistic regression analysis of QRT-PCR results. AGPAT1, B2M, IER3, IL1B, and BASP1 mRNAs were identified as those providing the highest discriminatory power for OC diagnosis. The findings were hindered by the small sample size and the lack of benign tumor control group [93]. Yang et al., 2021, evaluated the same salivary mRNAs expression profile in a Chinese population of 140 OC patients and 140 control individuals. Concomitantly, the CEA protein concentration was measured in blood. With machine-learning methods, the authors identified a novel panel of disease predictors. The validation phase on an independent cohort of 60 OC patients and 60 healthy subjects resulted in 85.0% of sensitivity and 88.3% of specificity, while measuring CEA concentration in blood, and BASP1 and IER3 levels in saliva. The mRNAs of interest were preselected from Lee et al. [93] who collected samples from Korean rather than Chinese women [94]. Li et al., 2018, assessed the potential use of immediate early response gene X-1 (IEX-1) transcript as OC BM. In OC, IEX-1 is down-regulated, thereby acting as a tumor suppressor. Its expression was quantified in saliva and blood samples with RT-qPCR. Three groups of patients were involved in the study: 26 patients of epithelial ovarian cancer (EOC), 37 women with benign ovarian tumors (BOT), and 55 controls. IEX-1 expression in blood and saliva was lower in EOC compared to BOT and controls. No significant differences were found in IEX-1 expression between BOT and controls. Diagnostic efficacy of IEX-1 was high in blood and medium in saliva but, of interest here, the results showed that both biofluids are suitable for BM detection. Saliva might have a higher specificity but lower sensitivity in discriminating EOC from BOT [95].

Mass spectrometry (MS) proved to be a powerful method to analyze fluids without extensive chemical preparation of samples. Tajmul et al., 2018, applied it in search for an OC signature in salivary proteins. On a proteome-wide scale, they analyzed quantitative differences in saliva using two-dimensional difference gel electrophoresis (2D-DIGE) combined with matrix-assisted laser desorption/ionization time of flight (MALDI-TOF) MS. They found 44 differentially expressed proteins, among which Lipocalin-2, indoleamine-2, 3-dioxygenase1 (IDO1), and S100A8 had the highest statistical scores. The three proteins, known to be involved in pathways of cancer progression and metastasis, were validated with Western blot and Elisa. Finally, salivary proteomics was combined with tissue-based transcriptomics. Immunohistochemistry, microarray, and QRT-PCR confirmed the up-regulation of these three candidate proteins in ovarian tissue. This signature holds a good diagnostic potential even though it would require validation in a larger population set [96].

Zermeño-Nava et al., 2018, applied surface-enhanced Raman spectroscopy (SERS) to measure levels of sialic acid (SA) in saliva samples collected from 37 women with benign ovarian adnexal masses (observed by TVS), and from 15 OC patients. Salivary SA levels were statistically different among the two groups, with a threshold corresponding to SA concentration > 15.5 mg/dL. However, SA cannot be considered a specific BM for OC: it is useful in the clinical scenario for diagnosing patients with adnexal mass, but not for screening the general female population [97].

Bel’skaya et al., 2019, used infrared (IR) Fourier spectroscopy to assess changes of the lipid profile in saliva in endometrial and OC patients. Three groups of women were involved: 51 ovarian and endometrial cancer patients, 26 positive controls of non-ovarian or endometrial cancer patients, and 30 healthy women. The authors identified a significantly reduced intensity of the absorption bands 2923/2957 cm^−1^ in OC and endometrial cancer patients [98].

Despite the methodological limitations due to the small size of patient groups, these works suggest promising directions for developing new diagnostic methods. The idea behind them all was to reduce unnecessary biopsies using non-invasive, saliva-based protocols for the early detection of OC [93].

Currently, studies exploring saliva for detection, diagnosis, or stratification of OC are quite heterogeneous. Transcriptomics and proteomics have been applied for an unbiased approach; however, some degree of partiality was still present in the selection of study participants. In addition, often the study design missed the positive control group (benign tumor or other cancer types). Further, discrepancies exist in the methods for processing samples (e.g., supernatant of saliva or whole saliva), all of which introduces some degree of fragmentation that hampers our comprehension of results. Finally, combining the analysis of saliva with other liquid or solid biopsies is certainly advantageous to better understand the disease; however, they are not so relevant in the search for effective BMs, regardless of their biological role.

## 7. Candidate miRNA Biomarkers of Ovarian Cancer in Other Biofluids

A multi-marker approach could improve the diagnosis of a heterogeneous disease such as OC, and microRNA are particularly suitable to build a multi-marker model. By improving methods and standardization procedures, miRNAs could become a routine tool to profile patients and select therapeutic interventions [99].

MiRNAs were characterized in biofluids of OC patients including blood (serum or plasma), urine, ascites, and utero-tubal lavage [100]. Currently, blood-derived biofluids still remain the gold standard for liquid biopsies; they have been extensively investigated in OC and in other oncological conditions as well. Here, we provide a comparison of the most representative results from a blood-based analysis and refer to other recent review articles for a more detailed knowledge of the literature [101,102,103].

### 7.1. Blood-Derived Biofluids

Several articles and systematic reviews have been written about OC and microRNAs in blood-derived biofluids since the diagnostic relevance of microRNAs detected in serum, plasma, and whole blood samples was reported by Resknick and colleagues for the first time in 2008 [104]. They profiled CF circulating miRNAs from serum samples of OC patients and healthy subjects. In the same year, Taylor and Taylor [43] focused on miRNAs content in OC-derived exosomes compared to benign controls in order to validate their potential as diagnostic BMs. Since then, several research groups have tried to find the most representative miRNA signature for pathogenesis of OC [100]. 

Hulstaert et al. in 2021, in a meta-analysis of the literature to identify candidate RNA BMs in body fluids for early diagnosis of OC, highlighted seventy-five RNAs candidates. Only ten of them were then considered good candidates because of their differential expression in at least two studies. Five miRNAs were up-regulated in OC patients compared to healthy subjects (miR-21, the miR-200 family, miR-205, miR-10a, and miR-346), and the other five (miR-122, miR-193a, miR-223, miR-126, and miR-106b) were down-regulated. Heterogeneity among all the reviewed publications was the main limitation for the authors’ work, especially concerning the methodologies [102]. 

Occasionally, some inconsistencies are found in the literature; they could result from relatively small size of samples, different ethnic groups recruited, or from the type of control groups (healthy subjects or benign disease patients) [103]. Additionally, pre-analytical parameters for sample preparation (blood volume, centrifugation speed, duration) can deeply affect the recovery of miRNAs from blood samples [102]. For example, plasma and serum undergo two distinct collection processes and they are differently affected by hemolysis. In plasma samples, coagulation is prevented by adding EDTA and, through centrifugation at high speeds for a long time, platelet-depleted plasma can be easily obtained. In turn, in serum samples, clot-activation causes the release of different biological molecules (also miRNAs) from platelets [105]. Even if, for the above reason, plasma should be considered the sample of choice to detect circulating CF-miRNAs, hemolysis can in both cases affect the accuracy of serum or plasma-based tests. Shah et al., 2016, evidenced the need of quantifying hemolysis as an essential step before measuring circulating miRNAs as potential diagnostic BMs [106].

Additionally, experimental approaches differ considerably among different studies: exosomal miRNAs are usually processed with ultracentrifugation methods or with specific isolation kits. Different methods could be also used for RNA isolation (extraction solutions with or without passage in columns) with varying performance. Then, among high-throughput methods, miRNAs can be profiled with microarrays or next-generation sequencing (NGS), followed by validation with QRT-PCR [100]. 

The initial screening analyses and criteria of miRNAs selection for subsequent validation differ among the reviewed articles. Three key examples follow. Kim et al. studied the expression level of seven exosomal miRNAs in the serum of OC patients. The seven candidates had been previously identified with high-throughput profiling studies as the most differentially expressed in OC tissues [107].

Elias et al. applied a neural network analysis to RNA-sequencing data from 179 serum samples. In an innovative way, they analyzed miRNA-seq data with a specific algorithm for discriminating OC patients from the other groups involved in the study [108].

Kumar et al. performed a miRNA screening analyzing the methylation status of genomic DNA. The differentially methylated regions of miRNA gene promoters led to identifying three hypomethylated regions through QRT-PCR on tissues and matched serum samples [109].

Another considerable constraint is represented by the use of endogenous controls for miRNA normalization, because currently the selection of reference genes has not yet been standardized. The most used spike-in control is cel-mir-39 which is added into the lysate, and its quantification used to evaluate the success of the isolation procedure. However, endogenous controls can also be used for circulating miRNAs, for example, small nuclear and nucleolar RNA such as U6, RNU44, RNU43, and RNU48. Several authors proposed other internal controls that were more stable in the biofluids of their interest, making comparisons across different articles even more difficult [105]. 

Another level of complexity is due to the dual possibility of isolating miRNAs as CF or as extracellular vesicle (EV)-contained molecules. In fact, EVs are released from many different cell types, not only cancer cells. Therefore, different extraction and detection methods might influence the efficacy of discriminating among normal- or cancer-derived EVs [110], with issues related to standard BM concentrations and small sample sizes [111]. In addition, most of the published studies on exosomal miRNAs do not use high-throughput discovery approaches as a first step. Instead, they mostly select candidate exosomal miRNAs from the literature and then proceed with validation or functional assays [105].

### 7.2. Other Biofluids

This review’s authors selected candidates miRNAs characterized in biofluids other than blood for OC liquid biopsy. 

Urine. Urinary miRNAs have been mostly explored in urological and gynecological diseases. As an advantage, urine is generated near to the OC site of origin and its collection is non-invasive [112]. Detectable miRNA level in urine is lower compared to blood. Probably most circulating miRNAs are reabsorbed by kidneys through a not yet clearly understood mechanism, or they are destroyed in the urinary tract by high levels of RNases [112]. In addition, some authors stressed that the supernatant fraction, but not the exosomal fraction, exhibit a diagnostic potential. Currently, there is not a consensus yet around this topic; it will be critical to define whether the exosomal or the supernatant fraction perform better to yield a representative signature of urinary miRNA [113,114].

Záveský et al., 2015, worked on miRNA in urine samples of pre/post-surgery endometrial and EOC patients. Analysis of the urine supernatant fraction show that miRNAs were differently expressed between pathological and healthy samples. On the contrary, while working with the exosomal RNAs, the authors did not find any difference for any miRNA under analysis [113,114].

Zhou et al., 2015, profiled miRNAs in the urine of three groups of subjects: ovarian serous adenocarcinoma patients (OSA), 26 patients with benign gynecological disease, and 30 healthy controls. They revealed a higher miR-30a-5p expression in ovarian serous adenocarcinoma patients (OSA) urines versus both controls and benign ovarian specimens. Further, comparing urine from OSA, GC, CRC, and healthy controls, they found miR-30a-5p upregulation in OSA and lower expression in the other cancer types, suggesting a diagnostic BM value for miR-30a-5p in OC. Moreover, the authors found miR-30a-5p upregulation in OSA tissue samples and cancer cell lines. Surgical removal of OSA affects urinary level of miR-30a-5p, further reinforcing the hypothesis of its ovarian origin [115].

Berner et al., 2022, detected differentially expressed miRNAs in urine, in OC cell culture, and cell culture supernatant. The authors confirmed the small total amount of miRNA that could be found in urine as one crucial issue for miRNA end-point usability [116]. Záveský et al., 2019, profiled the expression level of eight selected miRNAs, comparing tissue, ascites, and urine samples. Their work represented well the difficulties provided by urine. Some miRNAs were down-regulated in both ascitic fluid and tumor tissues in OC patients, while extracellular urine-derived miRNAs were not differentially expressed [117].

Ascites. Despite the fact that most of the studies are still based on RNA isolation from tissue, cell lines, or blood-derived biofluids, ascites is mentioned frequently in the scientific literature concerning OC, especially if compared with urine. In fact, more than other cancers, OC mostly disseminates in the peritoneal cavity, also because of the primary tumor site. The ascites, intraperitoneal liquid accumulation, is a symptom found mostly in OC patients at advanced stages, but sometimes it also occurs early on. It contains malignant tumor cells together with lymphocytes, mesothelial cells, macrophages, fibroblasts, and other cell types, that create a tumor microenvironment of soluble growth factors and cytokines. Tissue and ascites comparison for miRNAs expression level showed coherent results, confirming the close relation between the solid tumor and its dissemination in ascites [117]. Yet, the lack of systematic validationdoes not allow miRNA profiling from ascites as a novel diagnostic, prognostic, and predictive method. There are several problems related to the use of peritoneal effusions: (1) ascites (or effusion in general) is not always found in OC patients, especially at early stage; (2) effusion collection is an invasive procedure; (3) the components found in ascites are not evenly distributed, so variability between samples increases, making reproducibility even more difficult [118]; (4) there is still a gap in standardizing methods for sampling, separating extracellular fraction and isolating RNA; and (5) different studies employ different control groups (benign tumor lavages, plasma, or tissue of healthy controls) [119]. Ascites is considered an indicator of poor prognosis because it associates with tumor progression and chemoresistance [119].

Many studies have investigated miRNA expression level in ascites for the diagnosis of OC. Záveský et al., 2019, performed the first large-scale expression profiling of 754 miRNAs, working on ascitic fluid extracellular fraction (or ascites derived lavages) of high-grade serous OC patients and control plasma samples. After a screening phase with TaqMan array, seven miRNAs were validated with QRT-PCR. MiR-1290 and members of miR-200 family were found overexpressed in ascites compared to control plasma [119]. In a follow-up study, the same authors collected tissue samples and ascites, focusing on eight selected candidates (miR-203a-3p, miR-204-5p, miR-451a, miR-185-5p, miR-135b-5p, miR-182-5p, miR-200b-3p, and miR-1290) and validated six of them [117]. Yammamoto et al., 2018, examined miRNAs expression in EV from OC ascites, focusing on selected candidates. Six miRNAs were found significantly decreased in OC ascites compared to benign peritoneal fluids [120].

Vaksman et al., 2014, worked on exosomal miRNAs from effusion supernatants of OC patients. They started with a TaqMan array-based screening of miRNA from pooled exosomes derived from the effusion supernatants of OC patients, of tumor cells (positive control group), and of reactive mesothelium (negative control). 99 miRNAs showed higher expression in exosomes from OC effusion supernatants. MiR-21, miR-23a, and miR-29a were associated with poor prognosis. In vitro and in vivo assays were performed with selected miRNAs [121]. Similarly, Mitra and colleagues, 2021, characterized EVs from the ascites of OC patients to understand how EVs miRNA can influence growth, migration, and invasion in vitro and ex vivo assays. EVs from ascitic supernatants of patients with high-grade serous OC (HGSOC) or benign disease were profiled and then validated with QRT-PCR [122].

Wang and colleagues, 2022, identified an EV miRNA signature based on eight miRNAs (miR-1246, miR-1290, miR-483, miR-429, miR-34b-3p, miR-34c-5p, miR-145-5p, and miR-449a), analyzing plasma and ascites from malignant, high-grade serous OC patients and peritoneal fluids from benign gynecologic diseases patients. The authors showed how EV from malignant ascites increase the aggressive phenotypes of OC cells in 2D and 3D models. Gain and loss of function assays for the upregulated miR-1246 and miR-1290 showed their capacity of improving cell migration and invasiveness. Authors underlined the difficulties in obtaining ascites, and concomitantly to collect both plasma and ascites from the same patient [123].

Uterine cavity biofluids. The origin of high-grade serous carcinoma is still unclear. Since it could arise from the epithelium of the fallopian tube fimbriae, some authors explored the diagnostic value of utero-tubal lavage. This collection method allows to sample cells, or their secreted biological products, on the fimbriae. It is a safe and minimally invasive procedure: a saline solution is flushed in the uterine cavity and fallopian tube, then it is aspirated back from the gynecologic tract. Hulstaert et al., 2022, for the first time, sequenced the transcriptome of utero-tubal lavage and found upregulation of 300 mRNAs, mainly involved in cycle regulation and proliferation. Furthermore, they found 41 miRNAs more expressed in OC patients compared to healthy subjects. Five microRNAs were found to be associated to OC pathogenesis [124]. Skryabin et al., 2022, conducted the first exosomal miRNA profiling by small RNA-seq on uterine aspirates (UA) from EOC patients and healthy donors. The results showed significant differences for more than 57 miRNAs and three of them have been validated and confirmed for their up-/down- regulation in OC including miRNAs previously found associated with OC [125].

Proximal liquid biopsy and miRNAs detection remain insufficiently explored, although promising as a diagnostic method because they could be performed during routine gynecological visits. Moreover, the close relation with the site of origin of OC suggests the possibility to detect the disease when it is still at the initial stage. However, this is not applicable as a large-scale and rapid screening method because it is relatively time-consuming and requires specialized medical support.

To summarize the most relevant information, Table 2 reports an excursus on candidate miRNA BMs of OC in body fluids other than blood.

## 8. Conclusion and Future Perspectives

Current literature shows that saliva released by the major salivary glands contains various systemic BMs, thereby accurately reflecting pathophysiological conditions in humans. For this reason, salivary diagnostics were indicated by recent, authoritative editorials as a major resource for future diagnostics and for the low-cost screening procedures of systemic diseases including cancer [126].

MiRNAs present several advantages as possible BMs, over other molecules, for their accessibility, strong stability, and resistance to degradation in body fluids. More and more articles describe specific miRNA signatures in several tumor types [74]. Therefore, measuring quickly and accurately small variations of miRNAs concentration in body fluids may offer diagnostic and prognostic opportunities. International efforts aimed at a deeper understanding of circulating miRNA function and standardization of the miRNA analysis field (e.g., Extracellular RNA Communication Consortium, ERCC; https://exrna.org/ (accessed on 15 September 2022), or CANCER-ID), (www.cancerid.eu, accessed on 15 September 2022) [reviewed in Valihracha et al., 2019 [127], will help in providing more solid ground for biomedical exploitation of miRNAs.

Up to now, circulating miRNAs for OC detection have been mainly investigated in blood, a biofluid more complex than saliva in terms of collection, processing, and composition, where the high number of proteins represent a critical methodological step [128], as well as in other biofluids (Table 2). Salivary miRNAs, for their hitherto mentioned characteristics, might offer better performances compared to other fluids’ BMs [80].

In conclusion, the current lack of salivary tests for OC, whether based on miRNAs or other BMs, is a challenge to win so as to spare lives from OC and limit social costs. Current research efforts are ongoing to identify a pattern of salivary miRNAs suitable as BMs for OC diagnostics (D. Angeloni, personal communication). Auspicial results would make a possible alternative to the current options for excluding OC that still rely on gynecologist check-up, TVS, and blood test. Overall, this procedure could be expensive and difficult to manage, especially in remote areas or in disadvantaged socio-economic conditions.

For such a low-prevalence cancer type, a screening test would require a sensitivity for asimptomatic women > 75% and a specificity > 99.6% [129]. The currently used biomarkers for the blood test of OC are CA125 and HE4. They have been extensively studied and we refer to Dochez et al., 2019, for a summary of the sensitivity and specificity of the two protein biomarkers [130]. However, a pattern of good candidate miRNA BMs should reach at least the above suggested values.

With regard to the technological approach, the current on-chip technologies combined with photonic biosensors make it possible to envisage a strategy based on bringing together the molecular probes for detecting patterns of BMs within diagnostic devices that could speed up timing and reduce the costs of screening pertinent sections of the population to decrease mortality from OC [131]. Although such devices do not exist for clinical use yet, several proofs of concept exist, both for proteins [132,133] and RNA [134]. With further effort, one could imagine that combining the patterns of different BM types (e.g protein and RNA) could provide the screening robustness that single molecular types were not able to provide so far.

## Figures and Tables

**Table 2:** Biofluids other than blood or saliva for OC liquid biopsy and miRNAs BMs search (as selected by this review’s authors). EOC: FTC: fallopian tube cancer; HS: healthy subject; BGD: benign gynecological disease; AUC: area under the ROC curve. Each row provides information regarding the citation in parenthesis at the far left end of the row.

Reference	Biofluid	Source (Analytes)	miRNAs	Status	Case	Controls	AUC (95% CI)
[113]	urine	cell free	miR-92 miR-106b miR-100 miR-200b	↑ ↓ ↓ ↑	6 EOC FTC	13 HS	1.00 (0.815–1.000) 0.97 (0.764–1.000) 0.85 (0.601–0.970) 1.00 (0.782–1.000)
[115]	urine	exosomes	miR-30a-5p	↑	39 OSA	26 BGD 30 HS	0.86 (0.709–1.016)
[116]	urine	cell free	miR-15a let-7a	↑ ↓	13 OC	17 HS	
[117]	Ascite vs Plasma	exosomes	miR-203-3p miR-204-5p miR-135b-5p miR-182-5p miR-451a	↑ ↑ ↑ ↑ ↓	12 OC	12 HS	1.000 (0.782–1.000) 1.000 (0.782–1.000) 1.000 (0.782–1.000) 0.964 (0.725–1.000) 0.964 (0.725–1.000)
[120]	Ascite vs Benign peritoneal fluids	exosomes	let-7b. miR-23b miR-29a miR-30d miR-205 miR-720	↓ ↓ ↓ ↓ ↓ ↓	8 OC	10 HS	
[122]	Ascite and plasma	exosomes	miR-200c-3p miR-18a-5p miR-1246 miR-1290 miR-100- 5p miR125b-3p	↑ ↑ ↑ ↑ ↓ ↓	5 OC	2 BGD	
[123]	Ascite vs Benign peritoneal fluids	exosomes	miR-1246 miR-1290	↑ ↑	78 OC	72 BGD	
[124]	Uterine cavity fluids	Cell free	let-7d-5p miR-203a miR-200b miR-200c miR-191	↑ ↑ ↑ ↑ ↑	26 OC	48 BGD	
[125]	Uterine cavity fluids	exosomes	miR-451a miR-199a-3p miR-375-3p	↓ ↓ ↑	5 EOC	5 HS	

## Data Availability

This review article does not include original data. All statements are properly referred to citations.
